# Life expectancy, mortality risks and cause of death in patients with serious mental illness in South East London: a comparison between 2008–2012 and 2013–2017

**DOI:** 10.1017/S0033291721002257

**Published:** 2023-02

**Authors:** Chi-Kang Chang, Edward Chesney, Wei-Nung Teng, Sam Hollandt, Megan Pritchard, Hitesh Shetty, Robert Stewart, Philip McGuire, Rashmi Patel

**Affiliations:** 1 King's College London, Institute of Psychiatry, Psychology & Neuroscience, London, UK; 2 Department of Psychiatry, Taipei City Psychiatric Center, Taipei City Hospital, Taipei City, Taiwan; 3 South London and Maudsley NHS Foundation Trust, London, UK; 4 Department of Anaesthesiology, Taipei Veterans General Hospital and National Yang-Ming University, Taipei City, Taiwan

**Keywords:** Bipolar disorder, cause of death, life expectancy, mortality, schizophrenia, standardised mortality ratio

## Abstract

**Background:**

People with serious mental illness (SMI) have a significantly shorter life expectancy than the general population. This study investigates whether the mortality rate in this group has changed over the last decade.

**Methods:**

Using Clinical Record Interactive Search software, we extracted data from a large electronic database of patients in South East London. All patients with schizophrenia, schizoaffective disorder or bipolar disorder from 2008 to 2012 and/or 2013 to 2017 were included. Estimates of life expectancy at birth, standardised mortality ratios and causes of death were obtained for each cohort according to diagnosis and gender. Comparisons were made between cohorts and with the general population using data obtained from the UK Office of National Statistics.

**Results:**

In total, 26 005 patients were included. In men, life expectancy was greater in 2013–2017 (64.9 years; 95% CI 63.6–66.3) than in 2008–2012 (63.2 years; 95% CI 61.5–64.9). Similarly, in women, life expectancy was greater in 2013–2017 (69.1 years; 95% CI 67.5–70.7) than in 2008–2012 (68.1 years; 95% CI 66.2–69.9). The difference with general population life expectancy fell by 0.9 years between cohorts in men, and 0.5 years in women. In the 2013–2017 cohorts, cancer accounted for a similar proportion of deaths as cardiovascular disease.

**Conclusions:**

Relative to the general population, life expectancy for people with SMI is still much worse, though it appears to be improving. The increased cancer-related mortality suggests that physical health monitoring should consider including cancer as well.

## Introduction

Schizophrenia, schizoaffective disorder and bipolar disorder are associated with a life expectancy which is 10–20 years shorter than the general population (Hjorthøj, Stürup, McGrath, & Nordentoft, 2017). Almost all causes of death are elevated in these patients (Gatov, Rosella, Chiu, & Kurdyak, 2017), but 79% of the life expectancy gap is attributable to cardiovascular, respiratory, cancer, metabolic and infectious diseases (Jayatilleke et al., 2017). Causes more directly related to mental health problems, such as suicide, homicide and accidents, account for the remaining 21%.

Multiple factors appear to contribute to this reduction in life expectancy: increased rates of cigarette smoking, alcohol use and illicit substance use; poor diet and lack of exercise; adverse effects of antipsychotic and other medications; limited access to and engagement with physical and mental healthcare; and societal factors such stigma, social isolation, family breakdown and poverty (De Hert et al., 2011). With increasing awareness of the importance of physical healthcare in patients with serious mental illness (SMI), the pressure to intervene and improve outcomes has grown (De Hert et al., 2011). Moreover, it has been suggested that the size of the life expectancy gap may be increasing, as there is evidence that rates of smoking and deaths from cardiovascular disease are declining in the general population, but not in patients with SMI (Hayes, Marston, Walters, King, & Osborn, 2017; Royal College of Physicians; Royal College of Psychiatrists, 2013).

Over the last decade, there has been greater interest in the physical health of patients with SMI and interventions such as cardiovascular screening and smoke-free policies have been implemented (Huddlestone, Sohal, Paul, & Ratschen, 2018; Osborn et al., 2011). It is important to monitor mortality trends in these populations to assess whether changes in clinical practice are achieving their intentions. The present study sought to assess whether the mortality rate in patients with SMI from a well-characterised catchment area in South East London has changed over the last decade.

## Methods

### Study setting

Data were obtained from the South London and Maudsley (SLaM) National Health Service (NHS) Foundation Trust Biomedical Research Centre (BRC) Case Register. The database includes pseudonymised data for over 450 000 residents of four boroughs of London (Croydon, Lambeth, Lewisham and Southwark) who have received secondary mental healthcare since April 2006. The NHS provides mental healthcare for almost all patients with SMI in the UK; the private sector has a very limited role. Patients who have achieved sustained remission may be managed in primary care and would therefore not be included in this study.

### Cohort inclusion and exclusion criteria

We defined two observation periods: 1 January 2008 to 31 December 2012 and 1 January 2013 to 31 December 2017. These dates were chosen as they only include periods when the database was fully functional and they also allow sufficient time for cause of death data to be collected (as occasionally, in complex cases, there can be several years before an official cause of death is recorded). The final extraction for cause of death data was completed in April 2021. For each observation period, a cohort was defined. To be included in a cohort, participants had to receive a diagnosis of schizophrenia (F20), schizoaffective disorder (F25) or bipolar disorder (F31) according to the International Classification of Disease system 10th revision criteria (ICD-10) during that cohort's observation period. Diagnoses are assigned by SLaM clinicians at regular intervals, e.g. after outpatient clinical reviews, on referral to another team or on discharge from a ward. If an individual received more than one SMI diagnosis during an observation period, they were assigned the diagnosis they had received first. If a patient received a relevant diagnosis during both observation periods, they would be included in both cohorts. If a participant only received a relevant diagnosis during one of the two cohorts, and did not receive a relevant diagnosis during the other cohort, they were only included in the first cohort. The time when a participant entered into a cohort was defined as either the first ever SMI diagnosis or start of the time window (whichever occurs later). Exit from the cohort was defined as either the end of the time window or death (whichever occurs earlier). Patients without a recorded gender or date of birth, or who did not reach the age of 15 by the end of the study were excluded.

### Data extraction and mortality data

The Clinical Record Interactive Search (CRIS) software was used to extract data from the SLaM electronic health record (EHR) (Stewart et al., 2009). The software has an established record of enabling clinical data to be used for research purposes (Perera et al., 2016). Demographic and diagnostic information including gender, ethnicity, SMI diagnosis, diagnosis of alcohol (ICD: F10.x) or substance use disorder (ICD: F11.x-F19.x, excluding F17.x), date of diagnosis, date of birth, number of days as an inpatient during and number of admissions to a psychiatric hospital during each observational window was extracted. Apart from SMI diagnosis, all the above data are recorded in structured fields and are therefore not liable to data extraction errors. SMI diagnoses were also extracted from free-text fields, a method with established accuracy (Maudsley Biomedical Research Centre, 2021; Perera et al., 2016).

Deaths were identified by using the NHS Care Records Service, a nationwide service that records every death in the UK once a formal death certificate has been issued. Mortality data are updated in the EHR on a weekly basis using linked NHS numbers and included in the pseudonymised CRIS database. Data were obtained up until the end of the observation period. Cause of death data were obtained from a linked database of death certifications maintained and updated regularly by the UK Office of National Statistics (ONS) (Perera et al., 2016).

### Statistical analysis

#### Cohort comparison

The demographic and clinical characteristics of the two cohorts were compared using Student's *t* test for continuous variables and Pearson's χ^2^ test for categorical variables.

#### Standardised mortality ratios

Standardised mortality ratios (SMRs) were calculated by dividing the observed deaths in each population with the corresponding number of deaths expected from an equivalent age and gender standardised population with mortality rates from the general UK population (International Agency for Research on Cancer, 1980). Data on general population mortality risks across each study period were obtained from the ONS.

#### Period life expectancy

Chiang's II method of abridged life tables was used to calculate the estimates of life expectancy at birth according to gender and diagnosis (Chiang, 1984). The time that participants spent within each 5-year age band was summed to calculate the total person-years at risk for each age band. Data from individuals over the age of 85 were collated into a single ⩾85 years age band. Death rates (obtained from the ONS) for the general population were imputed for the 0–1, 1–4, 5–9 and 10–14-year age bands. If no deaths were recorded within 5-year age band, corresponding general population mortality risks were imputed. For the 2008–2012 cohort, ONS data from 2009–11 (the mid-point of the time window) were used. For the 2013–2017 cohort, ONS data from 2014–16 were used (Office for National Statistics, 2017).

The total person-years at risk and deaths for each age band were used to construct life tables to calculate life expectancy at birth. The life expectancy gap was calculated by subtracting the estimates from general population life expectancy from the periods 2009–11 (78.4 years for men; 82.4 years for women) and 2014–16 (79.2 years for men; 82.9 years for women), the mid-point of each cohort (Office for National Statistics, 2017). The life expectancy of patients with schizoaffective disorder was not calculated due to the small size of populations (<600).

#### Cause of death

Cause of death is defined in death certificates as the underlying disease or injury that initiates the train of morbid events leading directly to death. Recorded causes were grouped into the following ICD-10 categories:

- Natural causes:
  - cancer (C00-D48)
  - cardiovascular (I10-I99)
  - digestive (K00-K93)
  - metabolic (E00-E8890)
  - nervous and mental (F01-F99, G00-G98)
  - respiratory (J00-J99)
  - other natural causes (A00-B99; D50-D89);
- Unnatural causes
  - suicide (X60-X84, Y10-Y34, Y87.0)
  - other unnatural causes (S00-T88, U01, U02, U509, V00-W99, X0-X5, X85-X90, Y00-Y99 excluding Y87.0);
- No cause specified.

Cause of death was stratified according to gender and diagnosis. Pearson's χ^2^ test was used to compare differences between cohorts in the proportion of known causes of death (i.e. excluding ‘no cause specified’). Cause of death data were re-extracted in April 2021 to allow for delayed registrations. This led to a slight reduction in the size study population available (~3%) due to changes in local consent procedures.

Life tables were constructed using Microsoft Excel spreadsheets downloaded from the ONS (ONS life table template, 2016, 2017). All other analyses were completed using STATA (version 12.0)

#### Ethical approval

The SLaM BRC Case Register and CRIS have received ethical approval as an anonymised data set for secondary analyses from the Oxfordshire Research Ethics Committee C (18/SC/0372). The Clinical Data Linkage Service was used for data protection, linkage and extraction (Perera et al., 2016).

## Results

### Comparison between two cohorts

#### Demographic and clinical distribution

**Fig. 1.**
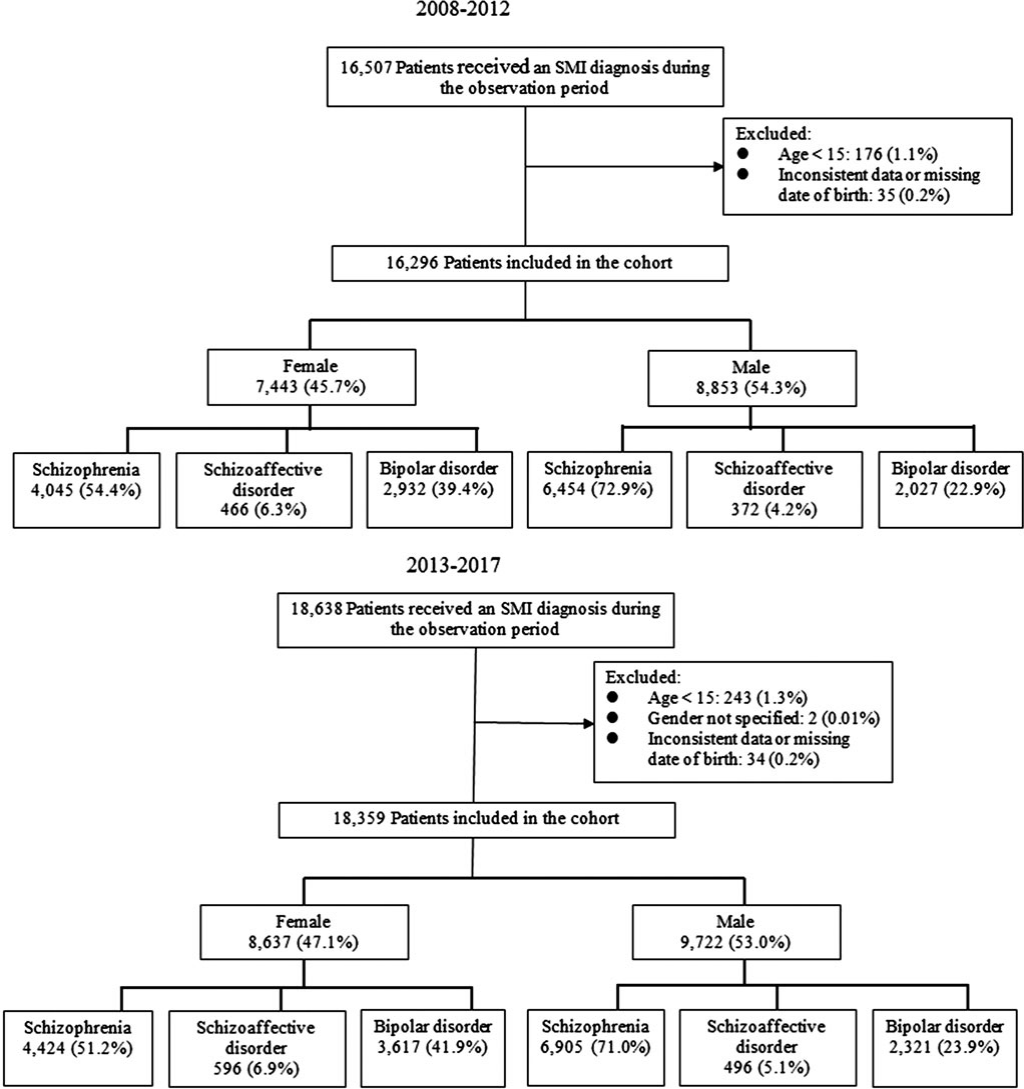
Flow chart. SMI, serious mental illness.

A total of 26 005 unique participants were included in the study. In total, 16 296 of these were in the early cohort and 18 359 were in the late cohort (Fig. 1). The demographic and clinical characteristics of each cohort are described in Table 1. There were small, statistically significant differences for several variables. In men, there was a statistically significant increase in mean age from 40.8 years (95% CI 40.5–41.1) in the early cohort to 41.8 years (95% CI 41.5–42.1) in the late cohort (*p* < 0.0001). For women, there was no significant change in mean age between cohorts: 44.0 years (95% CI 43.6–44.4) in the early cohort and 43.6 years (95% CI 43.3–44.0) in the late cohort (*p* = 0.213). For both genders, there were relatively more bipolar disorder diagnoses and fewer schizophrenia diagnoses in the late cohort (female: χ^2^ = 16.0, *p* < 0.001; male: χ^2^ = 12.2, *p* = 0.002). The mean follow-up time for individuals in the early cohort was 1292 days [standard deviation (s.d.): 607] compared to 1328 days (s.d.: 606) in the late cohort (*p* < 0.0001).

**Table 1.** Demographic and clinical characteristics of participants

		Early cohort (*n* = 8853)				Late cohort (*n* = 9722)			
Male		*n*	% Total	Deaths	% Group	*n*	% Total	Deaths	% Group
Age at entry	15	61	0.7%	0	0.0%	74	0.8%	0	0.0%
	15–24	1188	13.4%	18	1.5%	1207	12.4%	12	1.0%
	25–34	2147	24.3%	40	1.9%	2291	23.6%	53	2.3%
	35–44	2412	27.2%	94	3.9%	2286	23.5%	79	3.5%
	45–54	1597	18.0%	92	5.8%	2124	21.8%	122	5.7%
	55–64	796	9.0%	100	12.6%	992	10.2%	119	12.0%
	65+	652	7.4%	181	27.8%	748	7.7%	220	29.4%
Ethnicity	White	4512	51.0%	350	7.8%	4544	46.7%	363	8.0%
	Black	3134	35.4%	121	3.9%	3412	35.1%	161	4.7%
	Asian	251	2.8%	16	6.4%	265	2.7%	18	6.8%
	Other	544	6.1%	15	2.8%	695	7.1%	36	5.2%
	Not known	412	4.7%	23	5.6%	806	8.3%	27	3.3%
Marital status	Single	6703	75.7%	349	5.2%	7026	72.3%	407	5.8%
	Married	879	9.9%	62	7.1%	868	8.9%	68	7.8%
	Separated	813	9.2%	79	9.7%	755	7.8%	100	13.2%
	Not known	458	5.2%	35	7.6%	1073	11.0%	30	2.8%
Primary diagnosis	Schizophrenia	6454	72.9%	407	6.3%	6905	71.0%	447	6.5%
	Schizoaffective disorder	372	4.2%	14	3.8%	496	5.1%	23	4.6%
	Bipolar disorder	2027	22.9%	104	5.1%	2321	23.9%	135	5.8%
Alcohol use disorder	Yes	500	5.6%	40	8.0%	509	5.2%	51	10.0%
	No	8353	94.4%	485	5.8%	9213	94.8%	554	6.0%
Substance use disorder	Yes	820	9.3%	30	3.7%	982	10.1%	38	3.9%
	No	8033	90.7%	495	6.2%	8740	89.9%	567	6.5%
Psychiatric admissions	0	6122	69.2%	415	6.8%	6935	71.3%	480	6.9%
	1	1506	17.0%	63	4.2%	1527	15.7%	72	4.7%
	2	564	6.4%	24	4.3%	596	6.1%	28	4.7%
	3+	661	7.5%	23	3.5%	664	6.8%	25	3.8%
Inpatient days	0	5560	62.8%	377	6.8%	6341	65.2%	445	7.0%
	<4 weeks	890	10.1%	37	4.2%	894	9.2%	64	7.2%
	4 weeks–6 months	1428	16.1%	60	4.2%	1553	16.0%	67	4.3%
	>6 months	975	11.0%	51	5.2%	934	9.6%	29	3.1%

		Early cohort (*n* = 7443)				Late cohort (*n* = 8637)			
Female		*n*	% Total	Deaths	% Group	*n*	% Total	Deaths	% Group
Age at entry	15	64	0.9%	0	0.0%	115	1.3%	1	0.9%
	15–24	918	12.3%	8	0.9%	1191	13.8%	11	0.9%
	25–34	1454	19.5%	13	0.9%	1739	20.1%	12	0.7%
	35–44	1797	24.1%	48	2.7%	1770	20.5%	49	2.8%
	45–54	1426	19.2%	70	4.9%	1716	19.9%	68	4.0%
	55–64	813	10.9%	79	9.7%	987	11.4%	93	9.4%
	65+	971	13.0%	230	23.7%	1119	13.0%	290	25.9%
Ethnicity	White	3935	52.9%	296	7.5%	4182	48.4%	330	7.9%
	Black	2482	33.3%	115	4.6%	2712	31.4%	137	5.1%
	Asian	215	2.9%	8	3.7%	265	3.1%	10	3.8%
	Other	474	6.4%	15	3.2%	735	8.5%	26	3.5%
	Not known	337	4.5%	14	4.2%	743	8.6%	21	2.8%
Marital status	Single	4304	57.8%	175	4.1%	4914	56.9%	222	4.5%
	Married	1340	18.0%	66	4.9%	1360	15.7%	86	6.3%
	Separated	1446	19.4%	177	12.2%	1352	15.7%	189	14.0%
	Not known	353	4.7%	30	8.5%	1011	11.7%	27	2.7%
Primary diagnosis	Schizophrenia	4045	54.3%	277	6.8%	4424	51.2%	338	7.6%
	Schizoaffective disorder	466	6.3%	32	6.9%	596	6.9%	37	6.2%
	Bipolar disorder	2932	39.4%	139	4.7%	3617	41.9%	149	4.1%
Alcohol use disorder	Yes	234	3.1%	7	3.3%	253	2.9%	18	7.1%
	No	7209	96.9%	441	6.1%	8384	97.1%	506	6.0%
Substance use disorder	Yes	299	4.0%	10	3.3%	361	4.2%	14	3.9%
	No	7144	96.0%	448	6.1%	8276	95.8%	510	6.2%
Psychiatric admissions	0	5245	70.5%	328	6.3%	6505	75.3%	411	6.3%
	1	1208	16.2%	66	5.5%	1160	13.4%	63	5.4%
	2	455	6.1%	32	7.0%	535	6.2%	21	3.9%
	3+	535	7.2%	22	4.1%	437	5.1%	29	6.6%
Inpatient days	0	4783	64.3%	298	6.2%	6014	69.6%	382	6.4%
	<4 weeks	764	10.3%	29	3.8%	771	8.9%	33	4.3%
	4 weeks–6 months	1304	17.5%	79	6.1%	1362	15.8%	72	5.3%
	>6 months	592	8.0%	42	7.1%	490	5.7%	37	7.6%

#### Deaths, life expectancy and SMRs

**Fig. 2.**
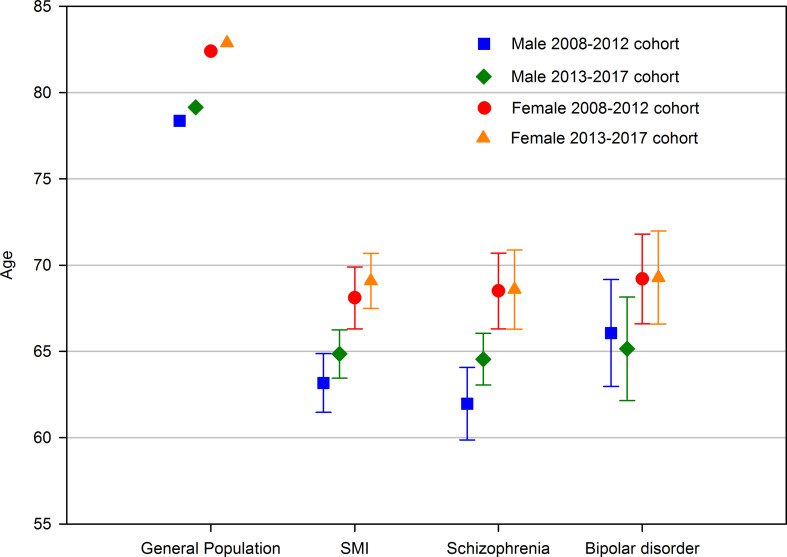
Life expectancy of each cohort, stratified by diagnosis and gender. SMI, serious mental illness.

The total number of deaths was 2102. Of these, 973 occurred between 2008 and 2012 (6.0% of cohort) and 1129 from 2013 to 2017 (6.1% of cohort). Table 1 describes the distribution of deaths according to demographic and clinical variables. Estimates of life expectancy at birth and the gap in life expectancy with the general population are described in Table 2 and Fig. 2. Life expectancy increased in male patients with any SMI diagnosis and schizophrenia but decreased in bipolar disorder. The greatest improvements were seen in men with schizophrenia (+2.5 years), though the confidence intervals of the two cohorts' estimates overlapped. In females, life expectancy increased for patients with any SMI diagnosis but there were only small changes in schizophrenia and bipolar disorder subgroups. SMRs are also described in Table 2. There were small changes for SMRs between cohorts which were in line with changes in life expectancy for each sub-group.

**Table 2.** Life expectancy and standardised mortality ratios

Males		Population (*n*)	Population-years (y)	Deaths (*n*)	SMR (95% CI)	LE at birth (y) (95% CI)	Change in LE between cohorts (y)	LE gap with general population (y)	Change in LE gap (y)
All SMI	2008–2012	8853	32 005	525	3.3 (3.0–3.6)	63.2 (61.5–64.9)		15.2	
	2013–2017	9722	35 955	605	3.2 (3.0–3.5)	64.9 (63.6–66.3)	+1.7	14.3	−0.9 (−5.9%)
Schizophrenia	2008–2012	6454	24 025	407	3.6 (3.2–3.9)	62.0 (59.9–64.1)		16.4	
	2013–2017	6905	26 098	447	3.3 (3.0–3.7)	64.6 (63.0–66.1)	+2.5	14.6	−1.7 (−10.6%)
Bipolar disorder	2008–2012	2027	6565	104	2.7 (2.1–3.2)	66.1 (63.0–69.2)		12.3	
	2013–2017	2321	7823	135	2.9 (2.4–3.3)	65.2 (62.3–68.2)	−0.9	14.0	+1.7 (+13.5%)
Schizoaffective disorder	2008–2012	372	1415	14	3.1 (1.5–4.7)	na		na	
	2013–2017	496	2034	23	3.0 (1.8–4.2)	na	na	na	na

Females		Population (*n*)	Population-years (y)	Deaths (*n*)	SMR (95% CI)	LE at birth (y) (95% CI)	Change in LE between cohorts (y)	LE gap with general population (y)	Change in LE gap (y)
All SMI	2008–2012	7443	25 499	448	2.8 (2.5–3.0)	68.1 (66.2–69.9)		14.3	
	2013–2017	8637	30 574	524	2.8 (2.5–3.0)	69.1 (67.5–70.7)	+1.0	13.8	−0.5 (−3.8%)
Schizophrenia	2008–2012	4045	14 322	277	2.6 (2.3–3.0)	68.5 (66.3–70.7)		13.9	
	2013–2017	4424	16 145	338	2.6 (2.3–2.9)	68.6 (66.3–70.9)	+0.1	14.3	+0.4 (+2.8%)
Bipolar disorder	2008–2012	2932	9427	139	2.9 (2.4–3.4)	69.2 (66.7–71.8)		13.2	
	2013–2017	3617	12 018	149	3.0 (2.5–3.5)	69.3 (66.6–72.0)	+0.0	13.6	+0.5 (+3.5%)
Schizoaffective disorder	2008–2012	466	1750	32	3.8 (2.5–5.1)	na		na	
	2013–2017	596	2411	27	3.5 (2.4–4.6)	na	na	na	na

SMI, serious mental illness; LE, life expectancy; SMR, standardised mortality ratio.

Comparisons are with ONS general population life expectancy estimates from the periods 2009–11 (78.4 years for men; 82.4 years for women) and 2014–16 (79.2 years for men; 82.9 years for women).

#### Cause of death

Out of 2042 deaths, 1950 (95.5% of total) had an ONS registered cause. These are described in Table 3. Due to delayed registration, 92 deaths (4.5% of total) had no specified cause. Cardiovascular causes were the most common cause of death (22.4%), followed by cancer (18.1%), respiratory disorders (14.5%), unnatural causes (13.1%), and nervous and mental disorders (10.8%). There were statistically significant differences in the distribution of known causes of death between cohorts for female patients with schizophrenia (χ^2^ = 16.8; *p* = 0.032). Cause of death data for patients with schizoaffective disorder are presented in the Supplementary materials.

**Table 3.** Cause of death by gender and diagnosis: 2008–2012 *v.* 2013–2017

	All patients		Schizophrenia		Bipolar disorder	
Males	2008–12	2013–17	2008–12	2013–17	2008–12	2013–17
Total	503	587	389	432	101	132
Natural deaths (*n*, %)	405 (81%)	466 (79%)	318 (82%)	348 (81%)	78 (77%)	102 (77%)
Cancer (*n*, %)	66 (13%)	108 (18%)	49 (13%)	84 (19%)	14 (14%)	21 (16%)
Cardiovascular (*n*, %)	113 (22%)	123 (21%)	88 (23%)	96 (22%)	24 (24%)	22 (17%)
Digestive (*n*, %)	37 (7%)	36 (6%)	29 (7%)	29 (7%)	8 (8%)	7 (5%)
Metabolic (*n*, %)	19 (4%)	14 (2%)	15 (4%)	10 (2%)	2 (2%)	3 (2%)
Nervous and mental (*n*, %)	43 (9%)	59 (10%)	33 (8%)	37 (9%)	10 (10%)	19 (14%)
Respiratory (*n*, %)	85 (17%)	79 (13%)	68 (17%)	60 (14%)	14 (14%)	17 (13%)
Other natural causes (*n*, %)	42 (8%)	47 (8%)	36 (9%)	32 (7%)	6 (6%)	13 (10%)
Unnatural deaths (*n*, %)	84 (17%)	90 (15%)	62 (16%)	57 (13%)	19 (19%)	28 (21%)
Accidental and other unnatural (*n*, %)	51 (10%)	68 (12%)	38 (10%)	43 (10%)	11 (11%)	20 (15%)
Suicide (*n*, %)	33 (7%)	22 (4%)	24 (6%)	14 (3%)	8 (8%)	8 (6%)
Difference in known causes of deaths between cohorts (χ^2^ test)	χ^2^ = 14.7, *p* = 0.064		χ^2^ = 13.7, *p* = 0.090		χ^2^ = 5.34, *p* = 0.720	
No cause specified (*n*, %)	14 (3%)	31 (5%)	9 (2%)	27 (6%)	4 (4%)	2 (2%)

	All patients		Schizophrenia		Bipolar disorder	
Females	2008–12	2013–17	2008–12	2013–17	2008–12	2013–17
Total	435	517	267	333	137	147
Natural deaths (*n*, %)	375 (75%)	437 (74%)	240 (62%)	287 (66%)	109 (108%)	120 (91%)
Cancer (*n*, %)	91 (18%)	104 (18%)	68 (17%)	67 (16%)	19 (19%)	26 (20%)
Cardiovascular (*n*, %)	116 (23%)	106 (18%)	79 (20%)	71 (16%)	31 (31%)	27 (20%)
Digestive (*n*, %)	25 (5%)	22 (4%)	13 (3%)	15 (3%)	10 (10%)	7 (5%)
Metabolic (*n*, %)	12 (2%)	18 (3%)	5 (1%)	10 (2%)	6 (6%)	7 (5%)
Nervous and mental (*n*, %)	42 (8%)	76 (13%)	27 (7%)	55 (13%)	12 (12%)	20 (15%)
Respiratory (*n*, %)	65 (13%)	68 (12%)	35 (9%)	40 (9%)	22 (22%)	21 (16%)
Other natural causes (*n*, %)	24 (5%)	43 (7%)	13 (3%)	29 (7%)	9 (9%)	12 (9%)
Unnatural deaths (*n*, %)	45 (9%)	48 (8%)	17 (4%)	27 (6%)	23 (23%)	18 (14%)
Accidental and other unnatural (*n*, %)	31 (6%)	29 (5%)	13 (3%)	15 (3%)	14 (14%)	11 (8%)
Suicide (*n*, %)	14 (3%)	19 (3%)	4 (1%)	12 (3%)	9 (9%)	7 (5%)
Difference in known causes of deaths between cohorts (χ^2^ test)	χ^2^ = 14.2, *p* = 0.077		χ^2^ = 16.9, *p* = 0.032		χ^2^ = 4.9, *p* = 0.768	
No cause specified (*n*, %)	15 (3%)	32 (5%)	10 (3%)	19 (4%)	5 (5%)	9 (7%)

## Discussion

Using a large clinical database from a secondary mental healthcare service in South East London, we found that patients with schizophrenia, schizoaffective disorder or bipolar disorder have a substantially lower life expectancy than the general population. Life expectancy in these patients appeared to improve between 2008–2012 and 2013–2017, such that the life expectancy gap relative to the general population was reduced by 0.9 years in men and 0.5 years in women, equivalent to 5.9% and 3.8% reductions in the size of the life expectancy gap. There was some variation within the diagnostic subgroups analysed. The greatest improvements were seen in men with schizophrenia where life expectancy increased by 2.5 years from 62.0 to 64.6 years, and the gap with the general population was reduced by 1.7 years (−10.6%). The most disappointing change was seen in men with bipolar disorder where life expectancy fell by 0.9 years from 66.0 to 65.2 years and the gap increased by 1.7 years (+13.5%). For each group analysed, similar changes were seen for SMRs.

In a systematic review of studies of life expectancy in schizophrenia, the weighted average of potential life years lost compared to the general population was 15.9 (95% CI 13.8–18.0) for men and 13.6 (95% CI 11.4–15.8) for women (Hjorthøj et al., 2017). These estimates are similar to the estimates of life expectancy gap in the present study (16.4 and 14.6 years in male cohorts; 13.9 and 14.3 in female cohorts). Chang et al. calculated life expectancy estimates for patients with SMI from South East London (Chang et al., 2011). They estimated a life expectancy at birth of 64.5 years for male patients and 69.9 years for female patients. These estimates are 1.3 and 1.8 years higher than those found in our first cohort. However, Chang et al. studied patients in a different observation window (2007–2009) which was also shorter than that used in our study (3 *v.* 5 years). Their study included significantly fewer deaths (446 *v.* 936) and is therefore likely to have calculated less precise estimates (Toson & Baker, 2003).

A number of previous studies have investigated changes in mortality risks in SMI populations. A meta-analysis of studies in schizophrenia stratified SMRs according to gender, diagnosis and time period (Oakley et al., 2018). In studies reporting data from 2000 to 2009, the SMRs were 3.5 (95% CI 3.3–3.8) for men and 3.5 (95% CI 3.0–4.0) for women. These fell to 2.8 (95% CI 2.3–3.5) for men and 2.7 (95% CI 2.1–3.4) for women in studies completed after 2010. Studies from the UK include Hoang et al., who investigated the mortality of patients with schizophrenia and bipolar disorder for a year after discharge from English hospitals between 1999 and 2006 (Hoang et al., 2011). They showed that age-standardised mortality ratios widened over time: from 1.6 (95% CI 1.5–1.8) in 1999 to 2.2 (2.0–2.4) in 2006 for patients with schizophrenia and from 1.3 (95% CI 1.1–1.6) in 1999 to 1.9 (95% CI 1.6–2.2) in 2006 for patients with bipolar disorder. A study by Hayes et al. measured mortality risks in schizophrenia and bipolar disorder from 2000 to 2014 using a nationally representative UK cohort of over 39 000 patients (Hayes et al., 2017). It demonstrated that the overall mortality rate for both diagnostic groups was decreasing but when compared to the general population, and after adjusting for a number of socio-demographic factors, the relative mortality risks had increased.

Together these studies would suggest that while there have been improvements in mortality risks for most populations of patients with SMI, the difference with the general population remains unacceptably large. Our study is consistent with these data and also raises concerns that the improvements in mortality are less evident in some subgroups, particularly male patients with bipolar disorder. Over recent years there has been increased awareness of the life expectancy gap with general populations and the importance of physical healthcare in SMI populations. Screening of cardiovascular risk in primary care has improved (Osborn et al., 2011), as has awareness of the physical health risks of certain antipsychotic medications (Correll, Detraux, De Lepeleire, & De Hert, 2015). One specific development which theoretically could have had the greatest impact was the introduction of UK ban on smoking in public places which was implemented in psychiatric hospitals in July 2008 and the subsequent expansion of smoking cessation services (Huddlestone et al., 2018). However, structural changes to NHS services (Lopez Bernal et al., 2017), particularly for the treatment of drug and alcohol problems (Oliver, 2018), and reduced number of psychiatric inpatient beds (Tyrer, Sharfstein, O'Reilly, Allison, & Bastiampillai, 2017) may have had a negative effect.

In this study, the most common causes of death were cardiovascular disease, respiratory disease and cancer, the same as those for the general population. Between cohorts, there were differences in the overall distribution of known causes of death for female patients with schizophrenia. In the 2013–2017 cohorts, cancer accounted for a similar proportion of deaths as cardiovascular disease in both men and women. A study of patients with mental illness from South East London suggested that elevated cancer mortality is ‘more likely to be accounted for by differences in survival after cancer diagnosis rather than by delayed diagnosis’ (Chang et al., 2014). In a US study of patients with breast cancer and schizophrenia, half had a disruption to their cancer care due to reasons such as psychiatric hospitalisation and refusal of care (Irwin, 2014). Future research should focus on ways in which patients with SMI and cancer could be supported to ensure that they receive an adequate level of care.

The strengths of this study include the large size of the cohorts sampled and the use of comprehensive nationwide mortality data, providing highly accurate data on deaths. Limitations include the incomplete registration of causes of death which affected the 2013–2017 cohort. The demographic and clinical characteristics of the patient populations changed between cohorts. This might have affected the results but though statistically significant, the sizes of the differences were small. Our analyses were stratified according to gender and life expectancy estimates take populations' age structure into account but other demographic or clinical variables were not adjusted for.

Life expectancy estimates, while providing an easily interpretable summary statistic of the overall mortality risks of a population, have received criticism due to the assumptions that they require (Perron, Simard, Brisson, Hamel, & Lo, 2017). Many patients with a diagnosis of schizophrenia or bipolar disorder do not receive a formal clinical diagnosis until after a period of assessment by mental health services. The early periods of a psychotic illness may present a particularly high risk of death for patients which are not accounted in our analysis. This introduces an immortal time bias and can lead to overestimation of life expectancy.

The generalisability of our results is limited as our data were collected from patients receiving secondary mental healthcare from the UK NHS, and not those managed in primary care services or the private sector. We only included patients from a single mental health trust in South East London, an urban area with high levels of ethnic diversity, socioeconomic inequality, substance use and social isolation. As a result, our findings may not be applicable to patients from other parts of the UK or from other countries.

In conclusion, over the last decade, there may have been small improvements in life expectancy and mortality risks for patients with SMI in South East London. However, in some subgroups, particularly male patients with bipolar affective disorder, life expectancy estimates fell and the gap with the general population increased. Cancer now accounts for a similar proportion of deaths as cardiovascular disease in both men and women. Future studies should continue to explore mortality, particularly in bipolar disorder, and explore what factors may account for less encouraging trends for these patients compared to those with schizophrenia. Healthcare services should continue to promote the importance of physical healthcare in SMI with particular emphasis on primary prevention of cardiovascular disease and cancer (Ilyas, Chesney, & Patel, 2017) and providing additional support for patients with serious physical illnesses.

## Supplementary material

For supplementary material accompanying this paper visit https://doi.org/10.1017/S0033291721002257. click here to view supplementary material
