# Supplementary material for: Life expectancy, mortality risks and cause of death in patients with serious mental illness in South East London: a comparison between 2008–2012 and 2013–2017
Source: Psychol Med. 2021 Sep 7;53(3):887–96. doi: 10.1017/S0033291721002257 (PMC9975985; doi:10.1017/S0033291721002257)
Supplement: Supplementary file 1 [file S0033291721002257sup001.docx]

## Supplementary Table 1 - Cause of death by gender and diagnosis: 2008-2012 versus 2013-2017, schizoaffective disorder and schizophrenia/schizoaffective disorder

| **Males** | **Schizoaffective disorder** |  | **Schizophrenia & Schizoaffective disorder combined** | |
| --- | --- | --- | --- | --- |
|  | **2008-12** | **2013-17** | **2008-12** | **2013-17** |
| **Total** | 13 | 23 | 402 | 455 |
| **Natural Deaths (n, %)** | 9 (69%) | 16 (70%) | 327 (81%) | 364 (80%) |
| **Cancer (n, %)** | 3 (23%) | 3 (13%) | 52 (13%) | 87 (19%) |
| **Cardiovascular (n, %)** | 1 (8%) | 5 (22%) | 89 (22%) | 101 (22%) |
| **Digestive (n, %)** | 0 (0%) | 0 (0%) | 29 (7%) | 29 (6%) |
| **Metabolic (n, %)** | 2 (15%) | 1 (4%) | 17 (4%) | 11 (2%) |
| **Nervous & Mental (n, %)** | 0 (0%) | 3 (13%) | 33 (8%) | 40 (9%) |
| **Respiratory (n, %)** | 3 (23%) | 2 (9%) | 71 (18%) | 62 (14%) |
| **Other natural causes (n, %)** | 0 (0%) | 2 (9%) | 36 (9%) | 34 (7%) |
|  |  |  |  |  |
| **Unnatural Deaths (n, %)** | 3 (23%) | 5 (22%) | 65 (16%) | 62 (14%) |
| **Accidental & Other Unnatural (n, %)** | 2 (15%) | 5 (22%) | 40 (10%) | 48 (11%) |
| **Suicide (n, %)** | 1 (8%) | 0 (0%) | 25 (6%) | 14 (3%) |
|  |  |  |  |  |
| **Difference in known causes of deaths between cohorts (chi-squared test)** | χ= 8.7 p = 0.277 | | χ= 14.6 p = 0.067 | |
|  |  |  |  |  |
| **No cause specified (n, %)** | 1 (8%) | 2 (9%) | 10 (2%) | 29 (6%) |
|  |  |  |  |  |
|  |  |  |  |  |
| **Females** | **Schizoaffective disorder** |  | **Schizophrenia & Schizoaffective disorder combined** | |
|  | **2008-12** | **2013-17** | **2008-12** | **2013-17** |
| **Total** | 31 | 37 | 298 | 370 |
| **Natural Deaths (n, %)** | 26 (200%) | 30 (130%) | 266 (66%) | 317 (70%) |
| **Cancer (n, %)** | 4 (31%) | 11 (48%) | 72 (18%) | 78 (17%) |
| **Cardiovascular (n, %)** | 6 (46%) | 8 (35%) | 85 (21%) | 79 (17%) |
| **Digestive (n, %)** | 2 (15%) | 0 (0%) | 15 (4%) | 15 (3%) |
| **Metabolic (n, %)** | 1 (8%) | 1 (4%) | 6 (1%) | 11 (2%) |
| **Nervous & Mental (n, %)** | 3 (23%) | 1 (4%) | 30 (7%) | 56 (12%) |
| **Respiratory (n, %)** | 8 (62%) | 7 (30%) | 43 (11%) | 47 (10%) |
| **Other natural causes (n, %)** | 2 (15%) | 2 (9%) | 15 (4%) | 31 (7%) |
|  |  |  |  |  |
| **Unnatural Deaths (n, %)** | 5 (38%) | 3 (13%) | 22 (5%) | 30 (7%) |
| **Accidental & Other Unnatural (n, %)** | 4 (31%) | 3 (13%) | 17 (4%) | 18 (4%) |
| **Suicide (n, %)** | 1 (8%) | 0 (0%) | 5 (1%) | 12 (3%) |
|  |  |  |  |  |
| **Difference in known causes of deaths between cohorts (chi-squared test)** | χ= 7.7 p = 0.463 | | χ= 13.1 p = 0.109 | |
|  |  |  |  |  |
| **No cause specified (n, %)** | 0 (0%) | 4 (17%) | 10 (2%) | 23 (5%) |
